# The Efficacy of Vaccination in the Prevention of Renal and Genital Leptospirosis in Experimentally Infected Sheep

**DOI:** 10.3390/tropicalmed7100321

**Published:** 2022-10-21

**Authors:** Gabriel Martins, Bruna Guadelupe, Luiza Aymée, Mario Felipe Alvarez Balaro, Pedro Henrique Pinto, Maria Isabel Nogueira Di Azevedo, Felipe Zandonadi Brandão, Walter Lilenbaum

**Affiliations:** 1 Laboratory of Veterinary Bacteriology, Biomedical Institute, Federal Fluminense University, Niterói 24210-030, RJ, Brazil; 2 Arthur Sá Earp Neto University Center, Petrópolis 25680-120, RJ, Brazil; 3 Pathology and Veterinary Clinics Department, Veterinary College, Federal Fluminense University, Niterói 24220-000, RJ, Brazil

**Keywords:** bacterins, pathogenic *Leptospira*, sheep, immunity

## Abstract

(1) Background: Leptospirosis, mainly determined by strains belonging to serogroup Sejroe, has a direct impact on the reproductive efficiency of ruminants, such as sheep. In Brazil, *Leptospira santarosai* serovar Guaricura, known to be able to chronically colonize the uterine environment, is of special note. Although vaccination minimizes the effects of acute disease, whether or not it can protect from renal colonization remains controversial, and its effects on the genital tract are unknown. In this context, the present study aims to investigate the efficacy of vaccination in the prevention of experimental genital leptospirosis. (2) Methods: Eighteen sheep were divided into three groups: one vaccinated with a polyvalent commercial bacterin, one vaccinated with an autologous bacterin, and one unvaccinated. After 14 days, the sheep were experimentally challenged with 10^8^ leptospires (*L. santarosai*, serogroup Sejroe, serovar Guaricura, strain FV52) intraperitoneally. Serology and colonization of the urinary and genital tracts were carried out monthly by PCR for 210 days in all animals. (3) Results: Vaccination significantly elicited antibody titers with a predominance of agglutinins against serogroup Sejroe. Both urinary and genital infections were confirmed by PCR. Positivity in urine PCR was absent after D120, while genital infection persisted until the end of the study. Serological results and the finding that vaccination did not avoid renal colonization align with previous studies. Despite vaccination, *Leptospira* established chronic and asymptomatic colonization of the genital tract until D210, an outstanding finding that remains to be fully understood in its mechanisms. (4) Conclusions: This is the first study conducted to analyze the effects of vaccination in the prevention of genital leptospirosis.

## 1. Introduction

Leptospirosis is an infectious disease caused by pathogenic strains of *Leptospira* sp. Immunity to leptospiral infection has been extensively studied; despite this, the understanding of protection mechanisms is still limited. Leptospirosis is typically a neglected disease, but also illustrates the One Health approach precisely. Initial immunity to leptospiral infection relies on the humoral defense and is mainly directed towards bacterial cell wall-LPS [1]. Interestingly, ruminants have distinct immunity mechanisms, and studies exploring the efficacy of vaccines in cattle have demonstrated that antibodies directed to lipopolysaccharide (LPS) may not be sufficient to protect against infection [2].

Several hosts have been designated as reservoirs of the bacterium, such as rodents for Icterohaemorrhagiae, dogs for Canicola, and ruminants for Hardjo, this last a member of serogroup Sejroe [2]. Strains of Hardjo have been described in the genital tracts of cows [3], genital discharges [4,5], oocytes [6], and, more recently, uteri [7,8] and follicular fluid [9,10]. In Brazil, *Leptospira santarosai* (strains of the serovar Guaricura, also from serogroup Sejroe) seem to be endemic in ruminants [11]. Besides having recovered from cervicovaginal mucus [5] and uteri [12] of asymptomatic naturally infected cows, the ability of these strains to colonize the uteri of experimentally infected hamsters [13] as well as sheep [14] has been demonstrated. Sheep have been described as a good experimental model to leptospirosis studies [14,15,16]. They are relatively small in comparison to cattle. Moreover, cattle and sheep have a strong chromosomal homology, which may justify the similarities in the immune responses that they present. This indicates that sheep are a good experimental model for evaluating the immune response of ruminants [15].

Once a herd is infected, *Leptospira* may persist for long periods [2]. Infected ruminants usually shed the agent through their urine, contaminating other animals and the environment. Additionally, the detection of leptospires in the genital tract reinforced the theory of the venereal transmission of leptospirosis [17]. In both cattle and small ruminants, infection mainly determines reproductive failures, such as infertility, abortion, stillbirth, weak offspring, and particularly estrus repetition, causing significant economic losses [18,19,20,21]. Recently, a review of the genital disease caused by leptospires in cattle suggested that it should be considered as a distinct syndrome, named bovine genital leptospirosis (BGL), which requires particular control mechanisms focused on the protection of the genital tract against colonization [22].

Given the above, vaccination is reported as an essential measure for the control of leptospirosis in animals, and its adoption in herds is widely recommended [23]. The efficacy of commercial vaccines in preventing renal colonization in cattle and small ruminants has been debated [24,25,26,27]. Nevertheless, their role in protecting the genital tract from leptospiral colonization has never been evaluated. Consequently, the present study aims to investigate the efficacy of vaccination in the prevention of experimental genital leptospirosis.

## 2. Materials and Methods

### 2.1. Study Design

Eighteen sheep belonging to the Santa Inês breed were divided into three groups (n = 6 each), two of them vaccinated, and one unvaccinated. After 14 days of the booster vaccination, the sheep were experimentally challenged with 10^8^ leptospires (*L. santarosai*, serogroup Sejroe, serovar Guaricura, strain FV52) intraperitoneally. Serology (MAT) and colonization of the urinary and genital tracts (by PCR) were carried out monthly for 210 days in all animals. DNA sequencing was carried out in PCR-positive samples to insure the leptospiral challenge. The study was conducted at the Laboratory of Veterinary Bacteriology and the Experimental Research Unit for Goats and Sheep (UniPECO) at the Federal Fluminense University school farm (http://labv.uff.br/unipeco (accessed on 16 October 2022). UniPECO adheres to current biosafety standards and is a Biosafety Level 2 (BSL-2) facility.

### 2.2. Animals

All 18 Santa Inês sheep were 12–14 months old, primiparous, and had never been vaccinated against leptospirosis. All animals were tested one week before the beginning of the study (urine PCR and serology) and showed to be free from leptospiral infection. The animals were randomly allocated to different groups using the Sorteio Rápido app (Xandroid, São Paulo, Brazil).

### 2.3. Experimental Groups

The 18 sheep were equally distributed into three groups. Groups A and B were vaccinated, and group C was the unvaccinated/control group. Group A was immunized intramuscularly (IM) with 2 mL of a commercial polyvalent vaccine. Group B was vaccinated with an autologous bacterin made from the same strain used in the experimental infection (FV52) and prepared according to the same protocol as that of the commercial vaccine. Both groups received the first dose 35 days before being experimentally challenged (D-35), and a second dose was delivered at D-14 (observing a 21-day interval between each dose, as recommended). Group C was inoculated with 1 mL of sterile physiological solution (0.9% NaCl) at the same instances.

Two weeks after the second immunization (D0), all animals were challenged with 1 mL of culture medium (EMJH), equivalent to 1 × 10^8^ leptospires, by the intraperitoneal route, as described by Rocha et al. [14]. All animals were monitored over a period of 210 days post-infection (p.i.). Throughout this period, clinical signs (fever, prostration, jaundice, hematuria, dyspnea, and dehydration) were monitored daily. Additionally, serological and molecular tests were conducted.

Throughout the study, none of the sheep displayed any apparent side effects and there were no indications of systemic disorders, such as jaundice or fever.

### 2.4. Vaccines

According to the manufacturer, the commercial vaccine used in this study is composed of strains of *Leptospira* spp. representing the serogroups Australis (serovar Bratislava), Canicola (serovar Canicola), Icterohaemorrhagiae (serovars Icterohaemorrhagiae and Copenhageni), Sejroe (serovars Wolffi, Hardjoprajitno, and Hardjobovis), and Pomona (serovar Pomona). Since MAT is only serogroup-specific, results are herein presented at a serogroup level. 

The autologous bacterin was prepared from inactivated leptospires of serogroup Sejroe, serovar Guaricura, strain FV52. Bacteria were cultivated for 10 days at 28 °C in an EMJH medium. The cultures were then centrifuged for one hour at 10,000 rpm/min and the sediment was resuspended in sterile PBS1x. The leptospires were counted in a Petroff-Hausser counting chamber, and the suspension was diluted to a concentration of 1 × 10^8^ spirochaetes per mL. The leptospires were inactivated with formalin at a final concentration of 0.3% divided into five 2 mL aliquots with aluminum potassium sulfate as an adjuvant at a final concentration of 10% (*v*/*v*). The final solution was maintained in a circular homogenizer for 24 h [28]. The dose used in each ewe was 1.0 mL inoculated intramuscularly in the region of the right flank. The vaccine was previously evaluated for purity, safety, and sterility according to CFR 113.105 [29].

### 2.5. Bacterial Strain

The FV52 strain was used in this experimental challenge. It was originally isolated from the cervicovaginal mucus of a cow in a slaughterhouse in Rio de Janeiro [11]. The bacterium belongs to the Collection of Bacterial Cultures of Veterinary Interest of the Laboratory of Animal Microbiology (http://labv.uff.br/ccbvet/ (accessed on 16 October 2022). The strain was characterized by serological and molecular methods as belonging to the *Leptospira santarosai* species and serogroup Sejroe. The bacterium was maintained in liquid nitrogen. To reactivate the virulence of the strain, leptospires were inoculated into hamsters and recovered from renal maceration in EMJH medium after death (7–10 days of incubation). After this, the isolate was transferred only once in EMJH medium for inoculum definition (10^8^ leptospires/mL). This strain was chosen because it has already demonstrated its ability to colonize the uteri of hamsters [13] and sheep [14].

### 2.6. Sampling

Blood (for serology), urine, and cervicovaginal mucus (CVM) (for PCR) samples were obtained when the first vaccine dose was administered (D-35), when the vaccine booster was administered (D-14), at D0 (experimental infection), and then monthly until D210 p.i. To obtain uterine fragments, a laparoscopic intervention was performed at D180 and D210 p.i.

Blood was collected by puncture of the jugular vein using sterile needles (40 × 12 mm) and was stored in vacuum tubes (without anticoagulant) (Vacutainer, BD, São Paulo, Brazil). Serum aliquots were stored in duplicates in 1.5 mL microtubes at a temperature of −20 °C until processing. Urine samples were collected by natural urination in conic sterile tubes (~15 mL) after the intravenous administration of furosemide 5 mg/kg (MSD, São Paulo, SP, Brazil). Additionally, CVM (vaginal fornix) was collected by the introduction of cytobrush (Vi-Pak, Copan Diagnostics, Murrieta, CA, USA) into the vaginal fornix and then stored in microtubes (1.5 mL) with 100 μL PBS 1× solution (DMPBS, Biodux, São Paulo, SP, Brazil). CVM samples were collected before urination to avoid contamination [22].

The ewes were deprived of food for 24 h and water for 12 h before laparoscopic intervention. Laparoscopies were carried out by placing the ewes in the dorsal recumbent position on a standard cradle for laparoscopic procedures. The surgical field, cranial to the udder, was shaved and disinfected. The procedure was carried out under sedation with acepromazine maleate IV (0.1 mg/kg; Acepran 1%, Vetnil, São Paulo, Brazil) and diazepam IV (0.3 mg/kg; UniDiazepax, União Química, São Paulo, Brazil). Subcutaneous local anesthetic (0.05 mL/kg of lidocaine; Lidovet, Bravet LDTA, Rio de Janeiro, Brazil) was injected at the trocar insertion. A pneumoperitoneum was induced through a cannula using a closed system with carbon dioxide, introducing approximately 2 L of inert air into the abdominal cavity. Subsequently, to visualize the uterus, a 5 mm 30 o endoscope (Karl Storz^®^, Tuttlingen, Germany) attached to a video camera was inserted into the abdominal cavity through a trocar approximately 5 cm cranial to the udder and 5 cm to the left of the midline. The uterus was handled using Babcock atraumatic forceps (33533BL Karl Storz^®^, Tuttlingen, Germany) through a second trocar placed approximately 5 cm cranial to the udder and 5 cm to the right of the midline. In sequence, a trucut biopsy needle (14 G) was inserted approximately 2 cm cranial to the right trocar, and one sample from the uterine body and another from the uterine horn (middle part) were collected. Following the laparoscopic process, all animals were treated with a single dose of meloxicam IM (2.0 mg/Kg; Maxican, Ouro Fino, São Paulo, Brazil).

For statistical analysis, the PCR results of CVM and the two uterine fragments were used. Positivity in any of the three samples indicated the status of the ewe as colonized by leptospires on the genital tract. 

### 2.7. Laboratory Procedures

#### 2.7.1. Serology (MAT)

Live cultures of ten strains of pathogenic *Leptospira* were used as antigens. These were representative of the serovars presented in the commercial vaccine (Bratislava, Canicola, Copenhageni, Hardjobovis, Hardjoprajitno, Icterohaemorrhagiae, Pomona, Wolffi) and two additional strains of the serovar Guaricura: M4/84 (reference strain) and FV52 (used in experimental infection). Antigens were maintained in a liquid EMJH medium, and testing was performed according to the standard procedure recommended by the World Organization for Animal Health [30]. Serum samples were first screened initially at a titer of 100 and those with an agglutination level > 50% were subsequently titrated against reacting antigens using serial twofold dilutions of serum. The endpoint was the highest tube in which 50% agglutination was recorded and measured by comparison with a control suspension. The same technician interpreted all reactions.

#### 2.7.2. Polymerase Chain Reaction (PCR)

Leptospiral DNA from the urine and CVM samples was extracted using the Wizard SV Genomic DNA Purification System (Promega, Madison, WI, USA). For uterine samples, DNA was extracted using the Qiagen DNeasy Blood & Tissue kit. Primers targeted the *lipL*32 gene (present in pathogenic leptospires), LipL32-45F (5′-AAG CAT TAC CGC TTG TGG TG-3′), and LipL32-286R (5′-GAA CTC CCA TTT CAG CGA TT-3′), which generate a fragment of 242 pb. Briefly, primers were used in concentrations of 0.6 µM, 1.0 U Taq polymerase, 2.4 µM MgCl_2_, and 0.3 mMdNTP in a final volume of 25 µL. The process was one cycle of initial denaturation at 94 °C for 2 min, followed by 35 cycles of denaturation at 94 °C for 30 s, annealing primers at 53 °C for 30 s, a 1-min extension at 72 °C, and a final extension cycle at 72 °C for 5 min. *Leptospira interrogans,* serovar Copenhageni, strain Fiocruz L1-130 (ATCCBAA-1198) was used as a positive control [9].

### 2.8. Data Analysis 

Data analysis was achieved using the SPSS Statistics 20 software package (IBM, Armonk, NY, USA). Graphics were created in GraphPad Prism 6 (GraphPad Software, La Jolla, CA, USA). Data from the titers observed in MAT and GIT were converted to log_10_ and treated by geometric means, as per Vallée et al. [31]. Additionally, animals with no antibody titers were excluded from the ANOVA. This correction ensured that the log title matched the tested dilution (1 to 1:100, 2 to 1:200, and 4 to 1:400). The means were calculated for each sampling by date and experimental group.

Non-parametric variables, such as seroreactivity (MAT) and PCR positivity, were assessed using the McNemar test (two samples) and the Cochran test (Q test) (K samples). The paired Wilcoxon test was applied to determine equality in the paired serology. Additionally, Fisher’s exact test was used to analyze 2 × 2 contingency tables at key points in the study to obtain serological data. 

Analyses were performed among vaccinated animals (groups A + B) in comparison with non-vaccinated animals (group C), between types of vaccines (groups A or B) in comparison with the control (group C), and between types of vaccine (group A × group B). All analyses were performed at a confidence level of 95% [32,33,34].

## 3. Results

### 3.1. Serological Results

At D35 (first vaccine dose), all sheep were seronegative. At D14, an important seroconversion (75%) was observed in both groups of vaccinated animals in comparison with the unvaccinated animals (*p* < 0.05). There was no significant difference between groups A and B, which indicates that both vaccines elicited a similar humoral response (*p* > 0.05).

**Figure 1:**
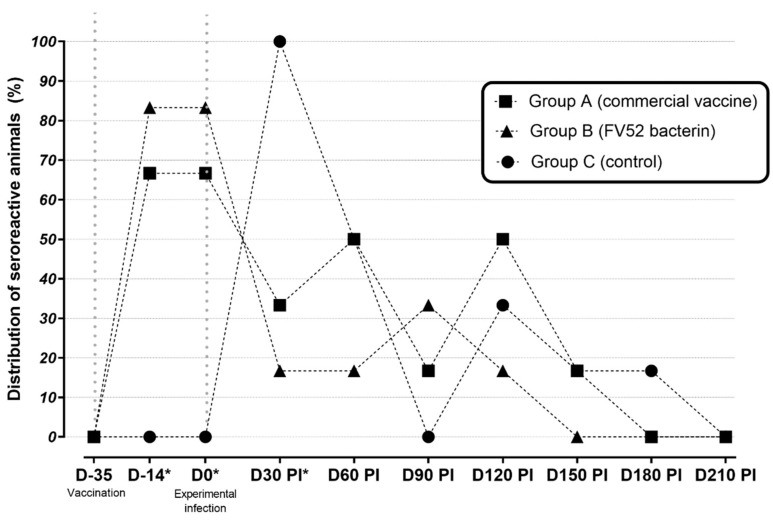
Percentage of seroreactive sheep as measured by MAT in unvaccinated (control) and vaccinated groups between D35 (first vaccine dose) and D210 post-infection. * *p* < 0.05 between vaccinated and non-vaccinated groups.

At D30 p.i., there was a significant reduction in the number of seroreactive animals in both vaccinated groups. At D60 p.i., no difference could be detected between the groups, and this remained the case until the end of the study (Figure 1).

Concerning the distribution of the serogroups, it was observed that the reactions to the serogroup Sejroe were significantly higher than those to other serogroups. This difference was observed at D14, D0, and D60 p.i. for group A; D14, and D0 for group B; and only at D30 p.i. for group C (Table 1).

There was an increase in antibody titers and seroreactive animals. Thus, in groups A and B, the highest antibody titers (≥200) were observed at D14 and D0. For the animals in group C, this occurred at D30 p.i. From this point, no further difference in antibody titers was observed.

### 3.2. Urinary PCR Results 

Regarding the detection of leptospiral DNA in urine, at D30 p.i., animals from all groups presented as renal carriers (67.7% in group A; 50% in group B, 67.7% in group C), confirming the infected status of the sheep. At D30 p.i., there was no difference in positivity in urinary PCR between the groups. From D120 p.i., positivity in urinary PCR was not observed in any animals from any group (Table 2).

### 3.3. Genital (CVM and/or Uterine Fragment) PCR Results

**Figure 2:**
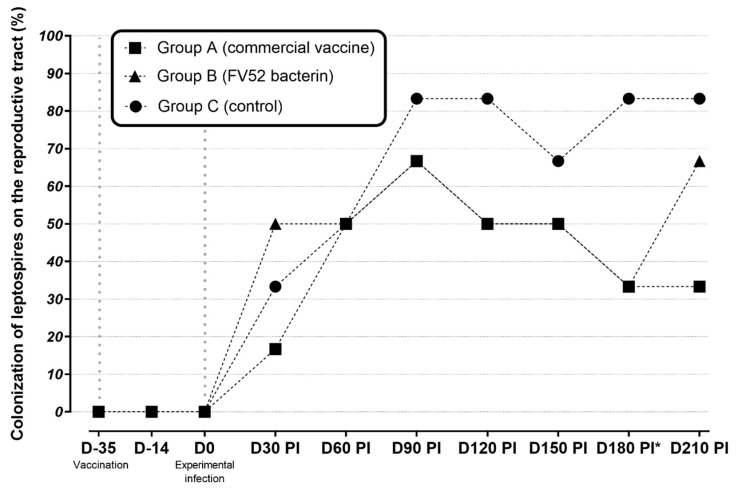
Distribution of leptospiral colonization (PCR) in the reproductive tracts of sheep in unvaccinated (control), vaccinated groups between D-35 (first vaccine dose) and D210 post-infection. * *p* < 0.05 between vaccinated and non-vaccinated groups.

At D30 p.i., leptospiral DNA in CVM was observed with similar PCR positivity found in all groups (16.7% in group A; 50% in group B, 33.3% in group C (*p* > 0.05)). The genital infection remained until the end of the study (D210 p.i.) (Figure 2). Regarding the presence of leptospires in the upper genital tract, uterine fragments from all groups were found to be positive. Group A presented 33.3% and 33.3%, group B presented 33.3% and 66.7%, and group C presented 16.7% and 33.3% of positivity at D180 and D210, respectively. 

## 4. Discussion

The serological results were not surprising and agree with the dynamics of the humoral response of ruminants after vaccination, which has been extensively studied [27,34]. Similarly to the present study, antibody titers have significantly decreased after 60 days post-vaccination to the same commercial vaccine used [34]. The similarity of the humoral response of naturally infected cows under field conditions and those of experimentally infected sheep is a desirable outcome since the reproducibility of outcomes reinforces the reliability of sheep as a good model for experimental studies on leptospirosis [16]. The first seroconversion, observed at D14, was a post-vaccinal response and for that reason, it did not appear in the unvaccinated sheep. This phenomenon was also observed by da Rosa et al. [15], who evaluated the immunogenicity of recombinant vaccines against leptospirosis in sheep. In their study, ewes also became seroreactive 1–2 weeks after vaccination. This fact highlights the ability of leptospiral antigens to incite the humoral immune response in sheep. Regarding the agglutinins observed after vaccination, the presence of anti-Sejroe antibodies was remarkable in the present study. This finding was also observed in naturally infected cows vaccinated against leptospirosis with the same commercial vaccine [34]. 

It is well-known that IgM appears soon after vaccination and also decreases fast. Thus, based on the observed long-term response, we assume that IgG was produced during the immunization process, as desired. Antibody titers were also observed among the unvaccinated animals later on, which probably happened as a response to the experimental infection, and these lasted until D30 when the three groups presented similar humoral responses. 

Kidney colonization has also been extensively studied [35,36], and once more, our findings align with those of previous studies. Urinary shedding was evident at D30 p.i. for all groups, indicating that the vaccines could not avoid kidney colonization. Nevertheless, that colonization apparently could not be maintained after D120, when no animals presented as renal carriers [37]. Other studies reported that vaccination cannot protect cattle from a kidney infection [38], which agrees with the present outcomes. In the present study, there was no significant difference between vaccinated and unvaccinated sheep in urine PCR. Although renal colonization disappeared after four months (for all groups), this was most likely due to the intrinsic characteristics of the infecting strain (Guaricura FV52), since studies conducted on hamsters [13] and sheep [14] had demonstrated the tendency of an auto-limited renal infection of that strain. Indeed, the pathogenesis of that strain herein observed was very similar to that observed by Rocha et al. [14]. In both studies, *Leptospira* established the infectious process in the kidneys and after drifted to the genital tract, remaining for a long term as chronic and asymptomatic. Although it was not possible to analyze the viability of *Leptospira* by culture or even to quantify its DNA, the positivity in end-point PCR in urinary and genital tract samples occurred for up to 120 days in the urine and 210 days in the genital samples. It suggests long-term maintenance of the bacteria in these systems, leading to a leptospiral shedding to the environment and also reinforcing the importance of the venereal transmission of leptospiral genital infection.

Regarding the main objective of the study concerning the novel topic of genital PCR positivity, the applied protocol, which was suggested by Rocha et al. [14], was successful in determining genital infection 30 days after experimental infection with Guaricura strain FV52. There was no significant difference (both in CVM and uterus samples) in the rate of genital colonization between the vaccinated and unvaccinated groups. It is difficult to determine how long that colonization could remain since the study was finalized seven months after the experimental infection when many animals were still infected. This indicates a persistent genital infection, which contrasts with the renal colonization, which was no longer detected after 30 days. Despite this finding, it is important to emphasize that the PCR results do not confirm the viability of the infective capacity of the bacteria, a topic that deserves to be elucidated in future studies. This is the first study conducted to analyze the efficacy of vaccination in the prevention of genital leptospirosis, so it is not possible to compare our outcomes with those of other authors. Although vaccination did not offer full protection from infection in the genital tract, a significant reduction in colonization at this site was observed in vaccinated animals 180 days after infection. The interaction of *Leptospira* and the renal/genital tract is complex and is a critical component that must be further explained [39]. In this context, we should have another look at how we assess the effectiveness of vaccines against leptospirosis in the genital tract. Assessment should also consider the pregnancy rate and capacity of ewes to carry a pregnancy to term.

For many years, genital tract infection has been considered a secondary effect of renal infection. However, it has recently been recognized as a distinct syndrome named bovine genital leptospirosis (BGL) [22]. Since the clinical aspects of bovine leptospirosis indicate that it is a reproductive disease associated with abortion, subfertility, and estrus repetition [21], very little is known about the efficacy of vaccines in preventing reproductive losses due to leptospiral infection [25]. Although a singular study has indicated that vaccines fail to confer protection and sterile immunity when animals were challenged with Hardjo (*L. borgpetersenii*) [40], more recent studies report a certain degree of reduction in the burden of the reproductive disease. Mughini-Gras et al. [32] report that vaccination before the breeding season can minimize reproductive losses, reflecting Pereira et al. [20] earlier findings. Indeed, even in preventing genital colonization, there is a gap in knowledge on the mechanism of vaccinations’ associations with reduction in reproductive losses. This is an important area of future research. 

## 5. Conclusions

This is the first study conducted to analyze the effects of vaccination in the prevention of genital leptospirosis. Extensive study is thus required to allow us to understand the mechanisms by which certain strains adapt to the genital tract and to help us to develop better tools for controlling genital leptospirosis, thus reducing reproductive and economic losses.

## Figures and Tables

**Table 1:** Percentage of seroreactivity of studied sheep as measured by MAT against serogroups included in the vaccine and experimental infection between D-35 (first vaccine dose) and D180 post-infection. * *p* < 0.05 between vaccinated and non-vaccinated groups.

DAY	GROUP	SEROGROUPS				
		Australis	Canicola	Icterohaemorrhagiae	Pomona	Sejroe
D-35	A (commercial vaccine)	0%	0%	0%	0%	0%
	B (FV52 bacterin)	0%	0%	0%	0%	0%
	C (control)	0%	0%	0%	0%	0%
D-14 *	A (commercial vaccine)	0%	0%	0%	16.7%	66.7%
	B (FV52 bacterin)	0%	0%	0%	0%	83.3%
	C (control)	0%	0%	0%	0%	0%
D0 *	A (commercial vaccine)	0%	0%	0%	16.7%	66.7%
	B (FV52 bacterin)	0%	0%	0%	0%	83.3%
	C (control)	0%	0%	0%	0%	0%
D30	A (commercial vaccine)	0%	0%	0%	0%	33.3%
	B (FV52 bacterin)	0%	0%	0%	0%	16.7%
	C (control)	0%	0%	0%	0%	100%
D60 *	A (commercial vaccine)	0%	0%	0%	0%	50%
	B (FV52 bacterin)	0%	0%	0%	0%	16.7%
	C (control)	16.7%	0%	0%	0%	33.3%
D90	A (commercial vaccine)	0%	0%	0%	0%	16.7%
	B (FV52 bacterin)	16.7%	0%	0%	0%	16.7%
	C (control)	0%	0%	0%	0%	0%
D120	A (commercial vaccine)	16.7%	0%	0%	0%	33.3%
	B (FV52 bacterin)	0%	0%	0%	0%	16.7%
	C (control)	0%	0%	0%	0%	33.3%
D150	A (commercial vaccine)	0%	0%	0%	0%	16.7%
	B (FV52 bacterin)	0%	0%	0%	0%	0%
	C (control)	0%	0%	0%	0%	16.7%
D180	A (commercial vaccine)	0%	0%	0%	0%	0%
	B (FV52 bacterin)	0%	0%	0%	0%	0%
	C (control)	0%	0%	0%	0%	16.7%

**Table 2:** Percentage of positive sheep in urinary PCR in non-vaccinated (control) and vaccinated groups between D-35 (first vaccine dose) and D210 post-infection. * *p* < 0.05 between vaccinated and non-vaccinated groups.

	Group A (Commercial Vaccine)	Group B (FV52 Bacterin)	Group C (Control)
D-35	0%	0%	0%
D-14	0%	0%	0%
D0	0%	0%	0%
D30 PI	66.7%	66.7%	50%
D60 PI	66.7%	33.3%	0%
D90 PI *	0%	66.7%	83.3%
D120 PI	0%	0%	0%
D150 PI	0%	0%	0%
D180 PI	0%	0%	0%
D210 PI	0%	0%	0%

## Data Availability

Not applicable.

## References

[1] Lata K.S., Vaghasia V., Bhairappanvar S., Patel S., Das J. (2020). Vaccine Design Against Leptospirosis Using an Immunoinformatic Approach. Immunoinformatics. Methods in Molecular Biology.

[2] Ellis W.A. (2015). Animal Leptospirosis. Curr. Top. Microbiol. Immunol..

[3] Ellis W.A., Thiermann A.B. (1986). Isolation of *Leptospira interrogans* Serovar Bratislava from Sows in Iowa. Am. J. Vet. Res..

[4] Dhaliwal G.S., Murray R.D., Ellis W.A. (1996). Reproductive Performance of Dairy Herds Infected with *Leptospira interrogans* Serovar Hardjo Relative to the Year of Diagnosis. Vet. Rec..

[5] Loureiro A.P., Pestana C., Medeiros M.A., Lilenbaum W. (2017). High Frequency of Leptospiral Vaginal Carriers among Slaughtered Cows. Anim. Reprod. Sci..

[6] Bielanski A., Surujballi O., Golsteyn Thomas E., Tanaka E. (1998). Sanitary Status of Oocytes and Embryos Collected from Heifers Experimentally Exposed to *Leptospira borgpetersenii* Serovar Hardjobovis. Anim. Reprod. Sci..

[7] Cabral Pires B., Berzin Grapiglia J., Moreira L., Jaeger L.H., Carvalho-Costa F.A., Lilenbaum W. (2018). Occurrence of Uterine Carriers for *Leptospira interrogans* on Slaughtered Cows. Microb. Pathog..

[8] Di Azevedo M.I.N., Pires B.C., Libonati H., Pinto P.S., Cardoso Barbosa L.F., Carvalho-Costa F.A., Lilenbaum W. (2020). Extra-Renal Bovine Leptospirosis: Molecular Characterization of the *Leptospira interrogans* Sejroe Serogroup on the Uterus of Non-Pregnant Cows. Vet. Microbiol..

[9] Di Azevedo M.I.N., Pires B.C., Barbosa L.F.C., Carvalho-Costa F.A., Lilenbaum W. (2021). Characterization of Leptospiral DNA in the Follicular Fluid of Non-Pregnant Cows. Vet. Rec..

[10] Dos Santos Pereira P.V., Di Azevedo M.I.N., Dos Santos Baptista Borges A.L., Loureiro A.P., Martins G., Carvalho-Costa F.A., Souza-Fabjan J.M.G., Lilenbaum W. (2022). Bovine Genital Leptospirosis: Evidence of Ovarian Infection by *Leptospira interrogans*. Vet. Microbiol..

[11] Loureiro A.P., Hamond C., Pinto P., Bremont S., Bourhy P., Lilenbaum W. (2016). Molecular Analysis of Leptospires from Serogroup Sejroe Obtained from Asymptomatic Cattle in Rio de Janeiro—Brazil Reveals Genetic Proximity to Serovar Guaricura. Res. Vet. Sci..

[12] Aymée L., Nogueira Di Azevedo M.I., de Souza Pedrosa J., de Melo J.D.S.L., Carvalho-Costa F.A., Lilenbaum W. (2022). The Role of *Leptospira santarosai* Serovar Guaricura as Agent of Bovine Genital Leptospirosis. Vet. Microbiol..

[13] Pinto P.S., Barbosa C., Ferreira A.M.R., Lilenbaum W. (2020). Short Communication: Uterine Leptospiral Infection Is Strongly Associated to Strains of Serogroup Sejroe on Experimentally Infected Hamsters. Microb. Pathog..

[14] Rocha B.R., Balaro M., Pereira P.V., Martins G., Lilenbaum W. (2018). Chronic Experimental Genital Leptospirosis with Autochthonous *Leptospira santarosai* Strains of Serogroup Sejroe. Small Rumin. Res..

[15] da Rosa M.C., Martins G., Rocha B.R., Correia L., Ferronato G., Lilenbaum W., Dellagostin O.A. (2019). Assessment of the Immunogenicity of the Leptospiral LipL32, LigAni, and LigBrep Recombinant Proteins in the Sheep Model. Comp. Immunol. Microbiol. Infect. Dis..

[16] Rocha B.R., Martins G., Lilenbaum W. (2020). An Historical View of the Experimental Leptospiral Infection in Ruminants. Comp. Immunol. Microbiol. Infect. Dis..

[17] Silva A.F., Farias P.J.A., Silva M.L.C.R., Araújo Júnior J.P., Malossi C.D., Ullmann L.S., Costa D.F., Higino S.S.S., Azevedo S.S., Alves C.J. (2019). High Frequency of Genital Carriers of *Leptospira* sp. in Sheep Slaughtered in the Semi-Arid Region of Northeastern Brazil. Trop. Anim. Health Prod..

[18] Ellis W.A., Bryson D.G., Neill S.D., McParland P.J., Malone F.E. (1983). Possible Involvement of Leptospires in Abortion, Stillbirths and Neonatal Deaths in Sheep. Vet. Rec..

[19] Leon-Vizcaino L., Hermoso de Mendoza M., Garrido F. (1987). Incidence of Abortions Caused by Leptospirosis in Sheep and Goats in Spain. Comp. Immunol. Microbiol. Infect. Dis..

[20] Pereira M.H.C., Cooke R.F., Alfieri A.A., Vasconcelos J.L.M. (2013). Effects of Vaccination against Reproductive Diseases on Reproductive Performance of Lactating Dairy Cows Submitted to AI. Anim. Reprod. Sci..

[21] Libonati H.A., Santos G.B., Souza G.N., Brandão F.Z., Lilenbaum W. (2018). Leptospirosis Is Strongly Associated to Estrus Repetition on Cattle. Trop. Anim. Health Prod..

[22] Loureiro A.P., Lilenbaum W. (2020). Genital Bovine Leptospirosis: A New Look for an Old Disease. Theriogenology.

[23] Martins G., Lilenbaum W. (2017). Control of Bovine Leptospirosis: Aspects for Consideration in a Tropical Environment. Res. Vet. Sci..

[24] Kasimanickam R., Whittier W.D., Collins J.C., Currin J.F., Inman B., Hall J.B., Pelzer K.D. (2007). A Field Study of the Effects of a Monovalent *Leptospira borgpetersenii* Serovar Hardjo Strain Hardjobovis Vaccine Administered with Oxytetracycline on Reproductive Performance in Beef Cattle. J. Am. Vet. Med. Assoc..

[25] Plunkett A.H., Graham T.W., Famula T.R., Oberbauer A.M. (2013). Effect of a Monovalent Vaccine against *Leptospira borgpetersenii* Serovar Hardjo Strain Hardjobovis on Fertility in Holstein Dairy Cattle. J. Am. Vet. Med. Assoc..

[26] Vallée E., Heuer C., Collins-Emerson J.M., Benschop J., Ridler A.L., Wilson P.R. (2018). Effects of Natural Infection by *L. borgpetersenii* Serovar Hardjo Type Hardjo-Bovis and *L. interrogans* Serovar Pomona, and Leptospiral Vaccination, on Sheep Growth. Prev. Vet. Med..

[27] Vallée E., Heuer C., Collins-Emerson J.M., Benschop J., Ridler A.L., Wilson P.R. (2018). Effects of Natural Infection by *L. borgpetersenii* Serovar Hardjo Type Hardjo-Bovis, *L. interrogans* Serovar Pomona and Leptospiral Vaccination on Sheep Reproduction. Theriogenology.

[28] Bolin C.A., Alt D.P. (2001). Use of a Monovalent Leptospiral Vaccine to Prevent Renal Colonization and Urinary Shedding in Cattle Exposed to *Leptospira borgpetersenii* Serovar Hardjo. Am. J. Vet. Res..

[29] 9 CFR 113.105—*Leptospira* Hardjo Bacterin. https://www.ecfr.gov/current/title-9/chapter-I/subchapter-E/part-113/subject-group-ECFR275e24cb93d1fab/section-113.105.

[30] World Organisation for Animal Health (OIE) (2012). Manual of Diagnostic Tests and Vaccines for Terrestrial Animals.

[31] Vallée E., Heuer C., Collins-Emerson J.M., Benschop J., Wilson P.R. (2015). Serological Patterns, Antibody Half-Life and Shedding in Urine of *Leptospira* Spp. in Naturally Exposed Sheep. N. Z. Vet. J..

[32] Mughini-Gras L., Bonfanti L., Natale A., Comin A., Ferronato A., La Greca E., Patregnani T., Lucchese L., Marangon S. (2014). Application of an Integrated Outbreak Management Plan for the Control of Leptospirosis in Dairy Cattle Herds. Epidemiol. Infect..

[33] Martins G., Loureiro A.P., Libonati H., Lilenbaum W. (2017). Humoral Response in Naturally Exposed Horses After Leptospiral Vaccination. J. Equine Vet. Sci..

[34] Martins G., Oliveira C.S., Lilenbaum W. (2018). Dynamics of Humoral Response in Naturally-Infected Cattle after Vaccination against Leptospirosis. Acta Trop..

[35] Dorjee S., Heuer C., Jackson R., West D.M., Collins-Emerson J.M., Midwinter A.C., Ridler A.L. (2009). Are White-Spot Lesions in Kidneys in Sheep Associated with Leptospirosis?. N. Z. Vet. J..

[36] Sanhueza J.M., Wilson P.R., Benschop J., Collins-Emerson J.M., Heuer C. (2018). Meta-Analysis of the Efficacy of *Leptospira* Serovar Hardjo Vaccines to Prevent Urinary Shedding in Cattle. Prev. Vet. Med..

[37] Gerritsen M.J., Koopmans M.J., Olyhoek T. (1993). Effect of Streptomycin Treatment on the Shedding of and the Serologic Responses to *Leptospira interrogans* Serovar Hardjo Subtype Hardjobovis in Experimentally Infected Cows. Vet. Microbiol..

[38] Bolin C.A., Thiermann A.B., Handsaker A.L., Foley J.W. (1989). Effect of Vaccination with a Pentavalent Leptospiral Vaccine on *Leptospira interrogans* Serovar Hardjo Type Hardjo-Bovis Infection of Pregnant Cattle. Am. J. Vet. Res..

[39] Putz E.J., Nally J.E. (2020). Investigating the Immunological and Biological Equilibrium of Reservoir Hosts and Pathogenic *Leptospira*: Balancing the Solution to an Acute Problem?. Front. Microbiol..

[40] Bolin C.A., Cassells J.A., Zuerner R.L., Trueba G. (1991). Effect of Vaccination with a Monovalent *Leptospira interrogans* Serovar Hardjo Type Hardjo-Bovis Vaccine on Type Hardjo-Bovis Infection of Cattle. Am. J. Vet. Res..

